# P044: MRSA-infection prevention potential in a 500-beds tertiary care hospital

**DOI:** 10.1186/2047-2994-2-S1-P44

**Published:** 2013-06-20

**Authors:** F Mattner, D Peter, C Weßels, S Messler

**Affiliations:** 1 Institut für Hygiene, Kliniken der Stadt Köln, Cologne, Germany

## Introduction

Many studies of infection prevention measures (ICM) appear proving a reduction of methicillin-resistant Staphylococcus aureus (MRSA) - colonisation or - infection rates. Infection control personal has to decide if ICM should be introduced in their hospital. Here we give an example how an infection prevention potential (IPP) could be calculated for MRSA patients in a 500-bed tertiary care hospital under a given MRSA-screening, contact isolation precaution and active surveillance program.

## Objectives

To estimate an additional IPP using results of recently published ICM, demonstrated by daily body washes with chlorhexidine (CHX) for the reduction of blood stream infections (BSI)( Climo MW et al. 2013 ), limited to *S. aureus* infections.

## Methods

MRSA screening policy included a general screening for all medical wards and the intensive care unit; in all other ward screening was performed as recommended by the Robert-Koch-Institut. A culture based screening (BD CHROMAgar MRSA II) was performed except PCR-based (Roche LightCycler®Advanced) screening in the intensive care unit. We reported results when MRSA was suspected. Distinct subgroups of MRSA patients were defined as I/II: colonisation on admission/nosocomial acquired colonisation without infection during hospital stay; III: nosocomial MRSA infection after colonisation status at least 3 days before infection. IV: nosocomial MRSA infection without prior colonisation, V: infection already present on admission.

## Results

In 2011 and 2012, I were 266/225, II: 19/30, III: 23/12, IV: 12/5. V: 25/18 patients, respectively. Preventable infections were III with 23 per 332 and 12 per 285 MRSA cases in 2011 and 2012 respectively, yielding a prevention potential of only 6,9% and 4,2% of all MRSA positive patients. There were 17 primary and secondary BSIs. Calculating with 8% reduction for *S. aureus* BSI by CHX body washes reported by Climo they would prevent only one case in our hospital within two years.

## Conclusion

Before introduction of new ICM proposed by new studies MRSA patients could be subgrouped and an IPP can be determined. Applying published reduction data on the in-house IPP numbers gives a first estimate if the new ICM could be a benefit.

## Disclosure of interest

None declared
